# Dimethyl and diethyl esters of 5,6-bis­(pyridin-2-yl)pyrazine-2,3-di­carb­oxy­lic acid: a comparison

**DOI:** 10.1107/S2056989016001080

**Published:** 2016-01-27

**Authors:** Montserrat Alfonso, Helen Stoeckli-Evans

**Affiliations:** a Institute of Chemistry, University of Neuchâtel, Av. de Bellevaux 51, CH-2000 Neuchâtel, Switzerland; b Insitute of Physics, University of Neuchâtel, rue Emile-Argand 11, CH-2000 Neuchâtel, Switzerland

**Keywords:** crystal structure, dimeth­yl, dieth­yl, di­carb­oxy­lic acid, pyrazine, pyridine, C—H⋯O and C—H⋯N hydrogen bonding

## Abstract

In dimethyl 5,6-bis­(pyridin-2-yl)pyrazine-2,3-di­carboxyl­ate, C_18_H_14_N_4_O_4_, (I), and diethyl 5,6-bis­(pyridin-2-yl)pyrazine-2,3-di­carboxyl­ate, C_20_H_18_N_4_O_4_, (II), the dimethyl and diethyl esters of 5,6-bis­(pyridin-2-yl)pyrazine-2,3-di­carb­oxy­lic acid, the orientation of the two pyridine rings differ. In (I), pyridine ring *B* is inclined to pyrazine ring *A* by 44.8 (2)° and the pyridine and pyrazine N atoms are *trans* to one another, while pyridine ring *C* is inclined to the pyrazine ring by 50.3 (2)°, with the pyridine and pyrazine N atoms *cis* to one another. In compound (II), the diethyl ester, which possesses twofold rotation symmetry, the pyridine ring is inclined to the pyrazine ring by 40.7 (1)°, with the pyridine and pyrazine N atoms *trans* to one another. In the crystal of (I), mol­ecules are linked by C—H⋯N hydrogen bonds, forming chains along [001]. The chains are linked by C—H⋯π inter­actions, forming a three-dimensional structure. In the crystal of (II), mol­ecules are linked *via* C—H⋯O hydrogen bonds, forming a three-dimensional framework. There are C—H⋯π inter­actions present within the framework.

## Abstract 2

In compound (I), the dimethyl ester of 5,6-bis­(pyridin-2-yl)pyrazine-2,3-di­carb­oxy­lic acid, pyridine ring *B* is inclined to pyrazine ring *A* by 44.8 (2)°. The N_pyrazine_—C—C—N_pyridine_ torsion angle is −133.7 (4)°, with the N atoms trans to each other. Pyridine ring *C* is inclined to pyrazine ring *A* by 50.3 (2)°. Here the N_pyrazine_—C—C—N_pyridine_ torsion angle is 50.7 (5)° and the N atoms are *cis* to one another. In compound (II), the diethyl ester, which possesses twofold rotation symmetry, the pyridine rings are inclined to the pyrazine ring by 40.7 (1)°, with the N atoms *cis* to one another.

## Chemical context   

5,6-Bis(pyridin-2-yl)pyrazine-2,3-di­carb­oxy­lic acid (**L1H_2_**) was synthesized to study its coordination behaviour with first row transitions metals (Alfonso, 1999 ▸). It exists as a zwitterion, with the adjacent pyridine and pyridinium rings almost coplanar due to the presence of an intra­molecular N—H⋯N hydrogen bond. The crystal structures of the zwitterion and different charged forms of **L1H_2_**, *viz.* the HCl, HClO_4_ and HPF_6_ salts, and details of the hydrogen bonding have been reported (Alfonso *et al.*, 2001 ▸).
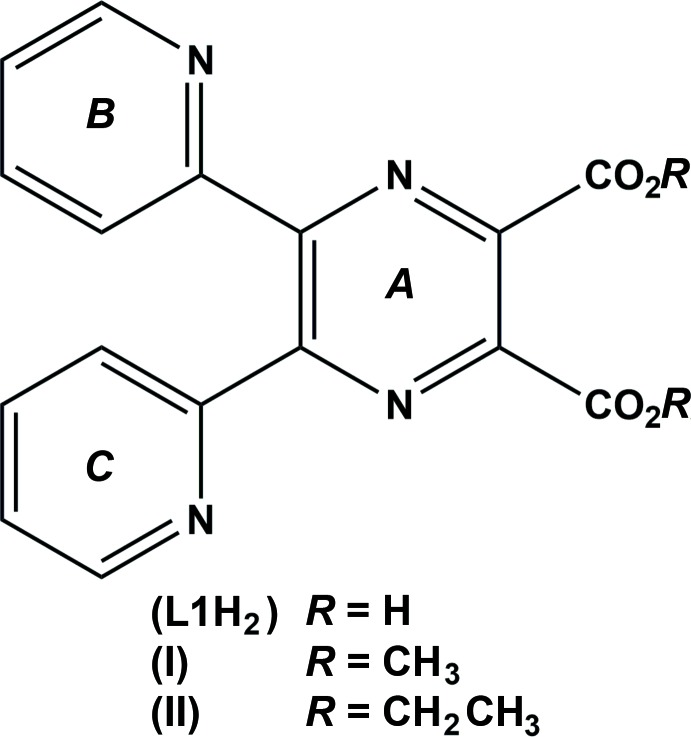


Metal-catalysed hydrolysis of amino acid esters is a well documented phenomenon (Dugas, 1989 ▸). It has been shown previously that the reaction of copper(II) salts with the dimethyl esters of pyrazine-2,3-di­carb­oxy­lic acid (Neels *et al.*, 1997 ▸) and 2,5-di­methyl­pyrazine-3,6-di­carb­oxy­lic acid (Wang & Stoeckli-Evans, 1998 ▸) resulted in the partial hydrolysis of the ligand and the formation of a two-dimensional network in the first case and a mononuclear complex in the second. Hence, metal-ion-promoted ester hydrolysis leads to the formation of new ligands and may serve as a general route to prepare new coordination compounds. The title compounds, (I) and (II), were synthesized to study the hydrolysis of these esters with first row transition metals (Alfonso, 1999 ▸), and we report herein on their syntheses and crystal structures.

## Structural commentary   

**Figure 1:**
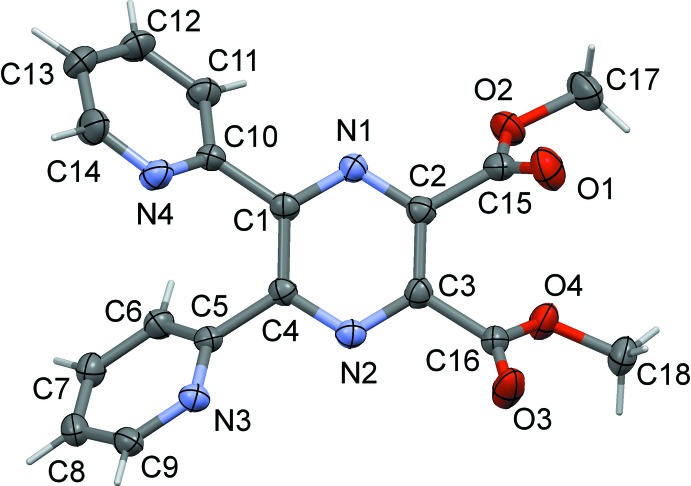
A view of the mol­ecular structure of compound (I), showing the atom labelling. Displacement ellipsoids are drawn at the 50% probability level.

As seen in compound (I), Fig. 1 ▸, the dimethyl ester of **L1H_2_**, pyridine ring *B* (N4/C10–C14) is inclined to the pyrazine ring (*A*; N1/N2/C1–C4) by 44.8 (2)° and the pyridine and pyrazine N atoms, N1 and N4, are *trans* to one another. Pyridine ring *C* (N3/C5–C9) is inclined to pyrazine ring *A* by 50.3 (2)°. However, here the pyridine and pyrazine N atoms, N2 and N3, are *cis* to one another. The two pyridine rings, *B* and *C*, are inclined to one another by 60.2 (2)°. The acetate groups, O1/O2/C15/C17 and O3/O4/C16/C18, are almost planar with r.m.s. deviations of 0.027 and 0.007 Å, respectively. They are inclined to the pyrazine ring by 60.3 (3) and 49.8 (3)°, respectively, and to one another by 42.4 (3)°.

**Figure 2:**
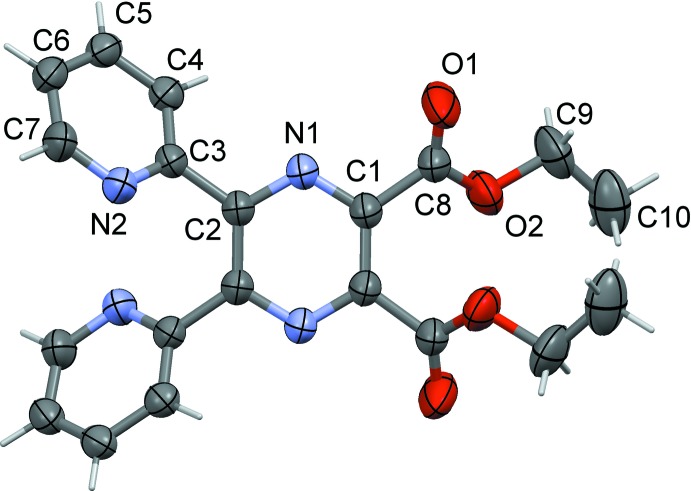
A view of the mol­ecular structure of compound (II), showing the atom labelling. Displacement ellipsoids are drawn at the 50% probability level. Unlabelled atoms are related to labelled atoms by the symmetry code (−*x* + 2, −*y* + 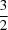, *z*).

Compound (II), the diethyl ester of **L1H_2_**, possesses twofold rotation symmetry, with the twofold rotation axis bis­ecting the C_ar_—C^i^_ar_ bonds [ar = aromatic; symmetry code (i): −*x* + 2, −*y* + 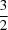, *z*], as shown in Fig. 2 ▸. The pyridine N atoms, N2 and N2^i^, face one another with an N2⋯N2^i^ separation of 3.043 (3) Å. The two pyridine rings are inclined to one another by 55.1 (1)° and to the pyrazine ring mean plane by 40.7 (1)°, with the pyrazine and pyridine N atoms, N1 and N2, *trans* to one another. The acetate group, O1/O2/C8/C9 [maximum deviation of 0.012 (3) Å for atom C8] is inclined to the pyrazine ring mean plane by 38.9 (1)°, and by 47.6 (2)° to the acetate group related by the twofold rotation axis. The oxygen atoms, O2 and O2^i^, are separated by only 2.840 (3) Å. The pyrazine ring in (II) has a slight twist-boat conformation (r.m.s. deviation = 0.046 Å) with the N1/C1/C2 and N1^i^/C1^i^/C2^i^ planes inclined to one another by 3.9 (3)°.

As noted above the differences in the structures of the two compounds lies essentially in the orientation of the pyridine rings with respect to the pyrazine ring (*cf* Figs. 1 ▸ and 2 ▸). It is possible that the slight distortion of the planarity of the pyrazine ring in (II), mentioned above, is related to the short N2⋯N2^i^ contact of 3.043 (3) Å of the adjacent pyridine rings and to the even shorter O2⋯O2^i^ contact of 2.840 (3) Å of the adjacent acetate groups**.**

## Supra­molecular features   

**Figure 3:**
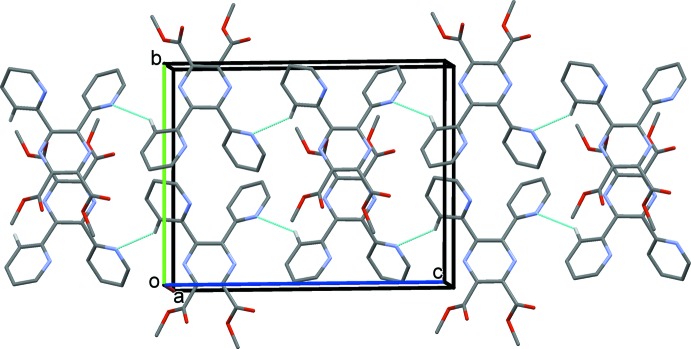
A view along the *a* axis of the crystal packing of compound (I). The hydrogen bonds are shown as dashed lines (see Table 1 ▸; only H atom H11 has been included).

In the crystal of (I), mol­ecules are linked by C—H⋯N hydrogen bonds, forming chains along [001]; see Table 1 ▸ and Fig. 3 ▸. The chains are linked *via* C—H⋯π inter­actions (Table 1 ▸), forming a three-dimensional structure.

**Figure 4:**
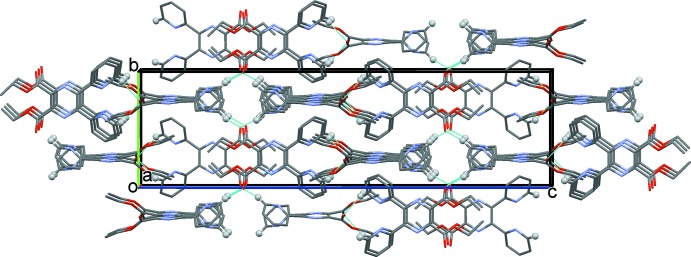
A view along the *a* axis of the crystal packing of compound (II). The hydrogen bonds are shown as dashed lines (see Table 2 ▸; only H atom H7 has been included).

In the crystal of (II), mol­ecules are linked *via* C—H⋯O hydrogen bonds, forming a three-dimensional framework; see Table 2 ▸ and Fig. 4 ▸. Within the framework there are a number of C—H⋯π inter­actions present (Table 2 ▸).

## Database survey   

Besides the structures of the zwitterion and different charged forms of **L1H_2_**, *viz.* the HCl, HClO_4_ and HPF_6_ salts (Alfonso *et al.*, 2001 ▸), the crystal structures of two copper(II) complexes of **L1H_2_** have been reported, *viz: catena*-[[[μ_3_-5,6-bis­(pyridin-2-yl)pyrazine-2,3-di­carboxyl­ate]tri­aqua­dibromo­dicopper(II)] methanol solvate trihydrate] and *catena*-[[[μ_4_-5,6-bis­(pyridin-2-yl)pyrazine-2,3-di­carboxyl­ate)di­aqua­dibromo­dicopper(II) monohydrate] (Neels *et al.*, 2003 ▸).

**Figure 5:**
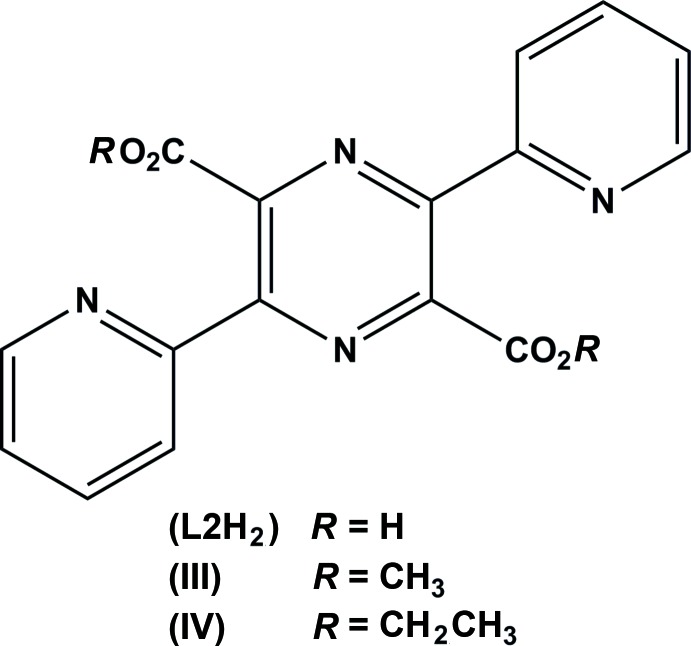
The chemical scheme for compound **L2H_2_**.

The structure of the isoelectronic compound 3,6-bis(pyridin-2-yl)pyrazine-2,5-di­carb­oxy­lic acid (**L2H_2_**), Fig. 5 ▸, has also been reported (Wang & Stoeckli-Evans, 2012*a* ▸). It too exists as a zwitterion and the structures of its di­hydro­chloride salt and the dimethyl sulfonate disolvate have also been reported (Wang & Stoeckli-Evans, 2012*a* ▸). The crystal structures of the dimethyl (III) and diethyl (IV) esters of **L2H_2_** have been deposited as private communications (Wang & Stoeckli-Evans, 2012*b* ▸,*c* ▸) with the Cambridge Structural Database (CSD; Groom & Allen, 2014 ▸). Both compounds crystallize in the triclinic space group *P* 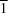 and possess inversion symmetry. The pyridine rings lie almost in the plane of the pyrazine ring and the N atoms are *trans* with respect to each other and to the nearest pyrazine N atom (as illustrated in Fig. 5 ▸). The ester groups are planar and in both compounds lie almost normal to the pyrazine ring. In the crystals of both compounds, inversion-related mol­ecules are linked *via* pairs of C—H⋯O hydrogen bonds, enclosing 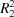
(10) ring motifs, forming chains propagating along [10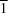].

## Synthesis and crystallization   

The synthesis of 5,6-bis­(pyridin-2-yl)pyrazine-2,3-di­carb­oxy­lic acid (**L1H_2_**) has been reported (Alfonso *et al.*, 2001 ▸). The dimethyl and diethyl esters, compounds (I) and (II), respectively, were obtained by the usual esterification procedure in acidic medium from the diacid and an excess of the corres­ponding alcohol.

**Synthesis of compound (I): dimethyl-5,6-bis­(pyridin-2-yl)pyrazine-2,3-di­carboxyl­ate L1H_2_**

(1.00 g, 3.11 mmol) was heated under reflux in freshly distilled MeOH (40ml) containing H_2_SO_4_ conc. (98%, 1 ml) during 16 h. After stopping the reaction, the temperature of the solution was allowed to cool to room temperature and then poured into an aqueous solution of NaOAc (6 g in 150 ml deionized water). The resulting solution was stirred in an ice bath containing NaCl to afford a white solid which was removed by filtration, washed with cold water and dried under vacuum. Single crystals suitable for X-ray analysis were obtained by the slow diffusion technique from CH_2_Cl_2_ and MeOH (yield: 0.77g, 65%; m.p. 410.2–411.7 K). Selected IR bands (KBr pellet, cm^−1^): ν = 1743(*s*), 1729(*vs*), 1339(*s*), 1302(*s*), 1283(*vs*), 1164(*s*), 1089(*vs*). ^1^H NMR (CDCl3, 400 MHz, p.p.m.): δ = 8.34(*dt*, 2H, *J* = 4.1Hz, *J* = 1.0 Hz, pyH), 7.99(*dt*, 2H, *J* = 7.7 Hz, *J* = 1.0 Hz, pyH), 7.82(*td*, 2H, *J* = 7.7 Hz, *J* = 1.0 Hz, pyH), 7.26(*td*, 2H, *J* = 7.7 Hz, *J* = 1.0 Hz, pyH), 4.04(*s*, 6H, CH_3_). ^13^C NMR (CDCl_3_, 50 MHz, p.p.m.): δ = 165.53, 155.98, 153.35, 149.26, 142.92, 137.64, 125.41, 124.49, 54.11. DCI–MS *m*/*z*: 351(*M*H^+^), 318, 279, 255, 208. Analysis for C_18_H_14_N_4_O_4_ (350.33), calculated C 61.70, H 4.04, N 15.99%, found C 61.4, H 3.91, N 15.65%.

**Synthesis of compound (II): diethyl-5,6-bis­(pyridin-2-yl)pyrazine-2,3-di­carboxyl­ate**

This compound was prepared by the same method as for (I). **L1H_2_** in freshly distilled EtOH containing catalytic amounts of H_2_SO_4_ conc. gave compound (II) as a white solid. Slow evaporation of an ethano­lic solution afforded colourless crystals suitable for X-ray analysis (yield: 0.70g, 62%; m.p. 390.5–391.3 K). Selected IR bands (KBr pellet, cm^−1^): ν = 3055(*w*), 1737(*s*), 1723(*vs*), 1368(*s*), 1301(*s*), 1276(vs), 1276(*vs*), 1161(*s*), 1086(*vs*). ^1^H NMR (CDCl_3_, 400 MHz, p.p.m.): δ = 8.33(*d*, 2H, *J* = 4 Hz, pyH), 8.01(*d*, 2H, *J* = 7.7 Hz, pyH), 7.81(*t*, 2H, *J* = 7.7 Hz, pyH), 7.24(*t*, 2H, *J* = 4.4 Hz, pyH), 4.52(*m*, 4H, *J* = 7 Hz, CH_2_), 1.45(*t*, 6H, *J* = 7.4 Hz, CH_3_). EI–MS *m*/*z*: 378 (34), 349 (9), 232 (95), 206 (66), 179 (25), 152 (11), 129 (9), 78(base), 46 (38). Analysis for C_20_H_18_N_4_O_4_ (378.38), calculated C 63.49, H 4.79, N 14.81%, found C 63.49, H 4.61, N 14.77%.

## Refinement   

Crystal data, data collection and structure refinement details are summarized in Table 3 ▸. For both compounds, the C-bound H-atoms were included in calculated positions and treated as riding atoms: C—H = 0.93–0.98 Å with *U*_iso_(H) = 1.5*U*_eq_(C-meth­yl) and 1.2*U*_eq_(C) for other H atoms. For compound (I), the Flack parameter (Parsons *et al.*, 2013 ▸) is = −0.2 (10), but it has no physical meaning here.

## Supplementary Material

Crystal structure: contains datablock(s) I, II, Global. DOI: 10.1107/S2056989016001080/gk2653sup1.cif

Structure factors: contains datablock(s) I. DOI: 10.1107/S2056989016001080/gk2653Isup2.hkl

Structure factors: contains datablock(s) II. DOI: 10.1107/S2056989016001080/gk2653IIsup3.hkl

Supporting information file. DOI: 10.1107/S2056989016001080/gk2653Isup4.cml

Supporting information file. DOI: 10.1107/S2056989016001080/gk2653IIsup5.cml

CCDC references: 1448182, 1448181

Additional supporting information:  crystallographic information; 3D view; checkCIF report

## Figures and Tables

**Table 1:** Hydrogen-bond geometry (Å, °) for (I) *Cg*2 is the centroid of the N3/C5–C9 pyridine ring.

*D*—H⋯*A*	*D*—H	H⋯*A*	*D*⋯*A*	*D*—H⋯*A*
C11—H11⋯N3^i^	0.93	2.57	3.334 (5)	140
C7—H7⋯*Cg*2^ii^	0.93	2.95	3.742 (5)	144
C17—H17*C*⋯*Cg*2^iii^	0.96	2.92	3.722 (6)	141

Symmetry codes: (i) 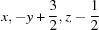; (ii) 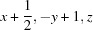; (iii) 
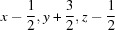
.

**Table 2:** Hydrogen-bond geometry (Å, °) for (II) *Cg*1 and *Cg*2 are the centroids of the pyrazine and pyridine rings N1/C1/C2/N1′/C1′/C2′ and N2/C3–C7, respectively [symmetry code (′): −*x* + 2, −*y* + 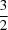, *z*].

*D*—H⋯*A*	*D*—H	H⋯*A*	*D*⋯*A*	*D*—H⋯*A*
C7—H7⋯O1^i^	0.94	2.48	3.308 (3)	147
C4—H4⋯*Cg*2^ii^	0.94	2.92	3.739 (2)	147
C10—H10*B*⋯*Cg*1^iii^	0.97	2.56	3.409 (3)	146
C10—H10*B*⋯*Cg*1^iv^	0.97	2.56	3.409 (3)	146

Symmetry codes: (i) 
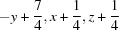
; (ii) 
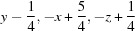
; (iii) 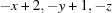; (iv) 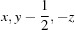.

**Table 3:** Experimental details

	(I)	(II)
Crystal data		
Chemical formula	C_18_H_14_N_4_O_4_	C_20_H_18_N_4_O_4_
*M*_r_	350.33	378.38
Crystal system, space group	Monoclinic, *I* *a*	Tetragonal, *I*4_1_/*a*
Temperature (K)	293	223
*a*, *b*, *c* (Å)	8.4249 (12), 12.2465 (10), 16.2561 (13)	10.2295 (6), 10.2295 (6), 36.281 (3)
α, β, γ (°)	90, 103.730 (8), 90	90, 90, 90
*V* (Å^3^)	1629.3 (3)	3796.5 (5)
*Z*	4	8
Radiation type	Mo *K*α	Mo *K*α
μ (mm^−1^)	0.10	0.10
Crystal size (mm)	0.70 × 0.50 × 0.38	0.65 × 0.50 × 0.50

Data collection		
Diffractometer	Stoe–Siemens AED2	Stoe IPDS 1
No. of measured, independent and observed [*I* > 2σ(*I*)] reflections	3035, 3028, 2737	14760, 1851, 1153
*R*_int_	0.012	0.043
(sin θ/λ)_max_ (Å^−1^)	0.606	0.616

Refinement		
*R*[*F* ^2^ > 2σ(*F* ^2^)], *wR*(*F* ^2^), *S*	0.050, 0.135, 1.11	0.049, 0.149, 1.01
No. of reflections	3028	1851
No. of parameters	238	129
No. of restraints	2	0
H-atom treatment	H-atom parameters constrained	H-atom parameters constrained
Δρ_max_, Δρ_min_ (e Å^−3^)	0.19, −0.21	0.34, −0.19

Computer programs: *STADI4* (Stoe & Cie, 1997 ▸), *EXPOSE*, *CELL* and *INTEGRATE* in *IPDS-I* (Stoe & Cie, 2004 ▸), *X-RED* (Stoe & Cie, 1997 ▸), *SHELXS97* (Sheldrick, 2008 ▸), *SHELXL2014* (Sheldrick, 2015 ▸), *PLATON* (Spek, 2009 ▸), *Mercury* (Macrae *et al.*, 2008 ▸), and *publCIF* (Westrip, 2010 ▸).

## supplementary crystallographic information

### (I) Dimethyl 5,6-bis(pyridin-2-yl)pyrazine-2,3-dicarboxylate Crystal data

**Table:**

C_18_H_14_N_4_O_4_	*F*(000) = 728
*M**_r_* = 350.33	*D*_x_ = 1.428 Mg m^−^^3^
Monoclinic, *I**a*	Mo *K*α radiation, λ = 0.71073 Å
*a* = 8.4249 (12) Å	Cell parameters from 33 reflections
*b* = 12.2465 (10) Å	θ = 14.1–19.6°
*c* = 16.2561 (13) Å	µ = 0.10 mm^−^^1^
β = 103.730 (8)°	*T* = 293 K
*V* = 1629.3 (3) Å^3^	Rod, colourless
*Z* = 4	0.70 × 0.50 × 0.38 mm

### (I) Dimethyl 5,6-bis(pyridin-2-yl)pyrazine-2,3-dicarboxylate Data collection

**Table:**

Stoe–Siemens AED2 diffractometer	θ_max_ = 25.5°, θ_min_ = 2.1°
ω/\2q scans	*h* = −10→10
3035 measured reflections	*k* = 0→14
3028 independent reflections	*l* = −19→19
2737 reflections with *I* > 2σ(*I*)	2 standard reflections every 60 min
*R*_int_ = 0.012	intensity decay: 1%

### (I) Dimethyl 5,6-bis(pyridin-2-yl)pyrazine-2,3-dicarboxylate Refinement

**Table:**

Refinement on *F*^2^	Secondary atom site location: difference Fourier map
Least-squares matrix: full	Hydrogen site location: inferred from neighbouring sites
*R*[*F*^2^ > 2σ(*F*^2^)] = 0.050	H-atom parameters constrained
*wR*(*F*^2^) = 0.135	*w* = 1/[σ^2^(*F*_o_^2^) + (0.0759*P*)^2^ + 1.0624*P*] where *P* = (*F*_o_^2^ + 2*F*_c_^2^)/3
*S* = 1.11	(Δ/σ)_max_ < 0.001
3028 reflections	Δρ_max_ = 0.19 e Å^−^^3^
238 parameters	Δρ_min_ = −0.21 e Å^−^^3^
2 restraints	Extinction correction: *SHELXL2014* (Sheldrick, 2015), Fc^*^=kFc[1+0.001xFc^2^λ^3^/sin(2θ)]^-1/4^
Primary atom site location: structure-invariant direct methods	Extinction coefficient: 0.0065 (18)

### (I) Dimethyl 5,6-bis(pyridin-2-yl)pyrazine-2,3-dicarboxylate Special details

**Table:**

Geometry. All e.s.d.'s (except the e.s.d. in the dihedral angle between two l.s. planes) are estimated using the full covariance matrix. The cell e.s.d.'s are taken into account individually in the estimation of e.s.d.'s in distances, angles and torsion angles; correlations between e.s.d.'s in cell parameters are only used when they are defined by crystal symmetry. An approximate (isotropic) treatment of cell e.s.d.'s is used for estimating e.s.d.'s involving l.s. planes.

### (I) Dimethyl 5,6-bis(pyridin-2-yl)pyrazine-2,3-dicarboxylate Fractional atomic coordinates and isotropic or equivalent isotropic displacement parameters (Å^2^)

**Table:**

	*x*	*y*	*z*	*U*_iso_*/*U*_eq_	
O1	0.8419 (5)	1.1583 (3)	0.0691 (2)	0.0470 (9)	
O2	1.0295 (4)	1.1079 (3)	−0.0014 (2)	0.0395 (8)	
O3	1.0067 (5)	1.1074 (3)	0.3016 (2)	0.0463 (10)	
O4	1.1454 (4)	1.1588 (3)	0.2054 (2)	0.0355 (8)	
N1	0.9519 (4)	0.8948 (3)	0.0567 (2)	0.0266 (8)	
N2	1.0688 (4)	0.8934 (3)	0.2330 (2)	0.0272 (8)	
N3	1.0738 (4)	0.6793 (3)	0.3087 (2)	0.0279 (8)	
N4	0.8579 (5)	0.6232 (3)	0.0803 (2)	0.0348 (9)	
C1	0.9822 (5)	0.8015 (3)	0.0992 (2)	0.0248 (9)	
C2	0.9784 (5)	0.9878 (3)	0.1014 (2)	0.0259 (9)	
C3	1.0319 (5)	0.9857 (3)	0.1896 (3)	0.0268 (9)	
C4	1.0489 (5)	0.8006 (3)	0.1880 (2)	0.0227 (8)	
C5	1.1158 (5)	0.6996 (3)	0.2355 (2)	0.0248 (9)	
C6	1.2218 (5)	0.6347 (4)	0.2045 (3)	0.0311 (9)	
H6	1.2486	0.6520	0.1537	0.037*	
C7	1.2879 (6)	0.5430 (4)	0.2500 (3)	0.0349 (10)	
H7	1.3595	0.4979	0.2303	0.042*	
C8	1.2459 (6)	0.5200 (3)	0.3248 (3)	0.0345 (10)	
H8	1.2879	0.4589	0.3565	0.041*	
C9	1.1394 (5)	0.5903 (4)	0.3517 (3)	0.0312 (9)	
H9	1.1118	0.5748	0.4025	0.037*	
C10	0.9393 (5)	0.6998 (3)	0.0486 (2)	0.0255 (9)	
C11	0.9763 (6)	0.6908 (4)	−0.0299 (3)	0.0334 (10)	
H11	1.0322	0.7464	−0.0499	0.040*	
C12	0.9291 (6)	0.5984 (4)	−0.0777 (3)	0.0389 (11)	
H12	0.9543	0.5900	−0.1301	0.047*	
C13	0.8443 (7)	0.5192 (4)	−0.0469 (3)	0.0413 (12)	
H13	0.8098	0.4561	−0.0779	0.050*	
C14	0.8113 (7)	0.5353 (4)	0.0320 (3)	0.0439 (13)	
H14	0.7529	0.4814	0.0524	0.053*	
C15	0.9399 (6)	1.0942 (4)	0.0546 (3)	0.0316 (10)	
C16	1.0580 (5)	1.0903 (4)	0.2399 (3)	0.0287 (9)	
C17	1.0131 (8)	1.2133 (4)	−0.0439 (3)	0.0525 (14)	
H17A	1.0924	1.2190	−0.0774	0.079*	
H17B	1.0307	1.2706	−0.0024	0.079*	
H17C	0.9053	1.2199	−0.0798	0.079*	
C18	1.1672 (6)	1.2674 (4)	0.2412 (3)	0.0386 (11)	
H18A	1.2114	1.3143	0.2049	0.058*	
H18B	1.2410	1.2645	0.2961	0.058*	
H18C	1.0636	1.2955	0.2464	0.058*	

### (I) Dimethyl 5,6-bis(pyridin-2-yl)pyrazine-2,3-dicarboxylate Atomic displacement parameters (Å^2^)

**Table:**

	*U*^11^	*U*^22^	*U*^33^	*U*^12^	*U*^13^	*U*^23^
O1	0.054 (2)	0.0354 (19)	0.054 (2)	0.0149 (17)	0.0183 (17)	0.0094 (17)
O2	0.055 (2)	0.0344 (19)	0.0313 (17)	−0.0011 (15)	0.0150 (15)	0.0089 (14)
O3	0.079 (3)	0.0309 (19)	0.037 (2)	−0.0093 (17)	0.0314 (19)	−0.0065 (14)
O4	0.0460 (18)	0.0266 (17)	0.0375 (16)	−0.0064 (13)	0.0171 (14)	−0.0077 (13)
N1	0.0339 (19)	0.0234 (19)	0.0235 (18)	−0.0009 (14)	0.0084 (14)	0.0004 (13)
N2	0.0320 (19)	0.027 (2)	0.0231 (18)	−0.0006 (14)	0.0081 (15)	−0.0003 (13)
N3	0.0337 (19)	0.0282 (19)	0.0223 (18)	−0.0009 (15)	0.0077 (14)	0.0010 (14)
N4	0.049 (2)	0.0326 (19)	0.0237 (17)	−0.0122 (17)	0.0103 (16)	−0.0023 (16)
C1	0.029 (2)	0.025 (2)	0.023 (2)	0.0003 (16)	0.0110 (17)	0.0009 (16)
C2	0.027 (2)	0.027 (2)	0.024 (2)	0.0006 (17)	0.0066 (16)	0.0011 (16)
C3	0.032 (2)	0.023 (2)	0.027 (2)	0.0005 (17)	0.0108 (18)	−0.0016 (16)
C4	0.0233 (19)	0.024 (2)	0.022 (2)	−0.0001 (15)	0.0082 (15)	0.0013 (15)
C5	0.029 (2)	0.024 (2)	0.0201 (19)	−0.0017 (16)	0.0032 (16)	−0.0009 (16)
C6	0.038 (2)	0.029 (2)	0.026 (2)	0.0039 (19)	0.0090 (18)	−0.0011 (18)
C7	0.040 (2)	0.027 (2)	0.036 (2)	0.0047 (19)	0.006 (2)	−0.0035 (19)
C8	0.043 (3)	0.022 (2)	0.034 (2)	0.0019 (19)	0.0007 (19)	0.0013 (18)
C9	0.038 (2)	0.029 (2)	0.024 (2)	−0.0046 (18)	0.0027 (18)	0.0040 (18)
C10	0.031 (2)	0.025 (2)	0.020 (2)	0.0015 (17)	0.0040 (16)	0.0015 (16)
C11	0.042 (3)	0.034 (2)	0.026 (2)	−0.0032 (18)	0.0130 (18)	−0.0015 (18)
C12	0.053 (3)	0.039 (3)	0.026 (2)	0.001 (2)	0.013 (2)	−0.007 (2)
C13	0.059 (3)	0.032 (3)	0.030 (2)	−0.003 (2)	0.003 (2)	−0.0083 (19)
C14	0.066 (3)	0.031 (2)	0.035 (3)	−0.018 (2)	0.012 (2)	0.002 (2)
C15	0.038 (2)	0.030 (2)	0.023 (2)	−0.002 (2)	0.0008 (17)	0.0001 (17)
C16	0.037 (2)	0.022 (2)	0.025 (2)	0.0026 (17)	0.0038 (17)	−0.0003 (17)
C17	0.069 (4)	0.039 (3)	0.045 (3)	−0.012 (3)	0.005 (3)	0.017 (2)
C18	0.047 (3)	0.024 (2)	0.046 (3)	−0.0043 (19)	0.013 (2)	−0.006 (2)

### (I) Dimethyl 5,6-bis(pyridin-2-yl)pyrazine-2,3-dicarboxylate Geometric parameters (Å, º)

**Table:**

O1—C15	1.202 (6)	C6—C7	1.387 (6)
O2—C15	1.324 (6)	C6—H6	0.9300
O2—C17	1.455 (5)	C7—C8	1.373 (7)
O3—C16	1.200 (6)	C7—H7	0.9300
O4—C16	1.325 (6)	C8—C9	1.387 (7)
O4—C18	1.446 (5)	C8—H8	0.9300
N1—C1	1.329 (5)	C9—H9	0.9300
N1—C2	1.340 (5)	C10—C11	1.388 (6)
N2—C3	1.331 (6)	C11—C12	1.377 (7)
N2—C4	1.341 (5)	C11—H11	0.9300
N3—C9	1.340 (5)	C12—C13	1.368 (7)
N3—C5	1.343 (5)	C12—H12	0.9300
N4—C10	1.335 (6)	C13—C14	1.389 (7)
N4—C14	1.336 (6)	C13—H13	0.9300
C1—C4	1.419 (5)	C14—H14	0.9300
C1—C10	1.489 (6)	C17—H17A	0.9600
C2—C3	1.397 (5)	C17—H17B	0.9600
C2—C15	1.505 (6)	C17—H17C	0.9600
C3—C16	1.508 (6)	C18—H18A	0.9600
C4—C5	1.495 (6)	C18—H18B	0.9600
C5—C6	1.377 (6)	C18—H18C	0.9600
			
C15—O2—C17	115.7 (4)	N4—C10—C11	123.2 (4)
C16—O4—C18	116.2 (3)	N4—C10—C1	117.0 (3)
C1—N1—C2	117.5 (3)	C11—C10—C1	119.7 (4)
C3—N2—C4	116.6 (3)	C12—C11—C10	119.1 (4)
C9—N3—C5	116.7 (4)	C12—C11—H11	120.5
C10—N4—C14	116.5 (4)	C10—C11—H11	120.5
N1—C1—C4	121.1 (4)	C13—C12—C11	118.8 (4)
N1—C1—C10	116.2 (3)	C13—C12—H12	120.6
C4—C1—C10	122.7 (4)	C11—C12—H12	120.6
N1—C2—C3	120.9 (4)	C12—C13—C14	118.3 (4)
N1—C2—C15	118.3 (3)	C12—C13—H13	120.9
C3—C2—C15	120.8 (4)	C14—C13—H13	120.9
N2—C3—C2	122.5 (4)	N4—C14—C13	124.2 (4)
N2—C3—C16	116.7 (3)	N4—C14—H14	117.9
C2—C3—C16	120.8 (4)	C13—C14—H14	117.9
N2—C4—C1	121.1 (4)	O1—C15—O2	125.6 (4)
N2—C4—C5	115.8 (3)	O1—C15—C2	122.9 (4)
C1—C4—C5	122.8 (4)	O2—C15—C2	111.6 (4)
N3—C5—C6	123.2 (4)	O3—C16—O4	126.2 (4)
N3—C5—C4	117.7 (3)	O3—C16—C3	124.5 (4)
C6—C5—C4	119.1 (4)	O4—C16—C3	109.4 (3)
C5—C6—C7	119.0 (4)	O2—C17—H17A	109.5
C5—C6—H6	120.5	O2—C17—H17B	109.5
C7—C6—H6	120.5	H17A—C17—H17B	109.5
C8—C7—C6	118.9 (4)	O2—C17—H17C	109.5
C8—C7—H7	120.5	H17A—C17—H17C	109.5
C6—C7—H7	120.5	H17B—C17—H17C	109.5
C7—C8—C9	118.3 (4)	O4—C18—H18A	109.5
C7—C8—H8	120.9	O4—C18—H18B	109.5
C9—C8—H8	120.9	H18A—C18—H18B	109.5
N3—C9—C8	123.9 (4)	O4—C18—H18C	109.5
N3—C9—H9	118.1	H18A—C18—H18C	109.5
C8—C9—H9	118.1	H18B—C18—H18C	109.5
			
C2—N1—C1—C4	−3.6 (5)	C5—N3—C9—C8	0.1 (6)
C2—N1—C1—C10	175.4 (3)	C7—C8—C9—N3	0.4 (7)
C1—N1—C2—C3	−1.6 (5)	C14—N4—C10—C11	−0.4 (7)
C1—N1—C2—C15	−178.2 (4)	C14—N4—C10—C1	176.0 (4)
C4—N2—C3—C2	−1.4 (5)	N1—C1—C10—N4	−133.7 (4)
C4—N2—C3—C16	−178.6 (4)	C4—C1—C10—N4	45.3 (5)
N1—C2—C3—N2	4.3 (6)	N1—C1—C10—C11	42.8 (5)
C15—C2—C3—N2	−179.2 (4)	C4—C1—C10—C11	−138.2 (4)
N1—C2—C3—C16	−178.6 (4)	N4—C10—C11—C12	−0.7 (7)
C15—C2—C3—C16	−2.1 (6)	C1—C10—C11—C12	−177.0 (4)
C3—N2—C4—C1	−3.8 (5)	C10—C11—C12—C13	1.2 (7)
C3—N2—C4—C5	170.5 (3)	C11—C12—C13—C14	−0.6 (8)
N1—C1—C4—N2	6.5 (5)	C10—N4—C14—C13	1.0 (8)
C10—C1—C4—N2	−172.4 (4)	C12—C13—C14—N4	−0.5 (8)
N1—C1—C4—C5	−167.4 (4)	C17—O2—C15—O1	4.4 (7)
C10—C1—C4—C5	13.7 (5)	C17—O2—C15—C2	−174.1 (4)
C9—N3—C5—C6	−0.6 (6)	N1—C2—C15—O1	120.0 (5)
C9—N3—C5—C4	−177.9 (4)	C3—C2—C15—O1	−56.6 (6)
N2—C4—C5—N3	50.7 (5)	N1—C2—C15—O2	−61.5 (5)
C1—C4—C5—N3	−135.1 (4)	C3—C2—C15—O2	121.9 (4)
N2—C4—C5—C6	−126.6 (4)	C18—O4—C16—O3	−6.2 (7)
C1—C4—C5—C6	47.6 (6)	C18—O4—C16—C3	173.9 (4)
N3—C5—C6—C7	0.7 (7)	N2—C3—C16—O3	−50.9 (6)
C4—C5—C6—C7	177.9 (4)	C2—C3—C16—O3	131.8 (5)
C5—C6—C7—C8	−0.2 (7)	N2—C3—C16—O4	128.9 (4)
C6—C7—C8—C9	−0.4 (7)	C2—C3—C16—O4	−48.3 (5)

### (I) Dimethyl 5,6-bis(pyridin-2-yl)pyrazine-2,3-dicarboxylate Hydrogen-bond geometry (Å, º)

Cg2 is the centroid of the N3/C5–C9 pyridine ring.

**Table:**

*D*—H···*A*	*D*—H	H···*A*	*D*···*A*	*D*—H···*A*
C11—H11···N3^i^	0.93	2.57	3.334 (5)	140
C7—H7···*Cg*2^ii^	0.93	2.95	3.742 (5)	144
C17—H17*C*···*Cg*2^iii^	0.96	2.92	3.722 (6)	141

Symmetry codes: (i) *x*, −*y*+3/2, *z*−1/2; (ii) *x*+1/2, −*y*+1, *z*; (iii) *x*−1/2, *y*+3/2, *z*−1/2.

### (II) Diethyl 5,6-bis(pyridin-2-yl)pyrazine-2,3-dicarboxylate Crystal data

**Table:**

C_20_H_18_N_4_O_4_	*D*_x_ = 1.324 Mg m^−^^3^
*M**_r_* = 378.38	Mo *K*α radiation, λ = 0.71073 Å
Tetragonal, *I*4_1_/*a*	Cell parameters from 5000 reflections
*a* = 10.2295 (6) Å	θ = 3.3–52.1°
*c* = 36.281 (3) Å	µ = 0.10 mm^−^^1^
*V* = 3796.5 (5) Å^3^	*T* = 223 K
*Z* = 8	Block, colourless
*F*(000) = 1584	0.65 × 0.50 × 0.50 mm

### (II) Diethyl 5,6-bis(pyridin-2-yl)pyrazine-2,3-dicarboxylate Data collection

**Table:**

Stoe IPDS 1 diffractometer	1153 reflections with *I* > 2σ(*I*)
Radiation source: fine-focus sealed tube	*R*_int_ = 0.043
Plane graphite monochromator	θ_max_ = 26.0°, θ_min_ = 2.1°
φ rotation scans	*h* = −12→12
14760 measured reflections	*k* = −12→12
1851 independent reflections	*l* = −44→44

### (II) Diethyl 5,6-bis(pyridin-2-yl)pyrazine-2,3-dicarboxylate Refinement

**Table:**

Refinement on *F*^2^	Secondary atom site location: difference Fourier map
Least-squares matrix: full	Hydrogen site location: inferred from neighbouring sites
*R*[*F*^2^ > 2σ(*F*^2^)] = 0.049	H-atom parameters constrained
*wR*(*F*^2^) = 0.149	*w* = 1/[σ^2^(*F*_o_^2^) + (0.0925*P*)^2^] where *P* = (*F*_o_^2^ + 2*F*_c_^2^)/3
*S* = 1.01	(Δ/σ)_max_ < 0.001
1851 reflections	Δρ_max_ = 0.34 e Å^−^^3^
129 parameters	Δρ_min_ = −0.19 e Å^−^^3^
0 restraints	Extinction correction: *SHELXL2014* (Sheldrick, 2015), Fc^*^=kFc[1+0.001xFc^2^λ^3^/sin(2θ)]^-1/4^
Primary atom site location: structure-invariant direct methods	Extinction coefficient: 0.0049 (10)

### (II) Diethyl 5,6-bis(pyridin-2-yl)pyrazine-2,3-dicarboxylate Special details

**Table:**

Geometry. All e.s.d.'s (except the e.s.d. in the dihedral angle between two l.s. planes) are estimated using the full covariance matrix. The cell e.s.d.'s are taken into account individually in the estimation of e.s.d.'s in distances, angles and torsion angles; correlations between e.s.d.'s in cell parameters are only used when they are defined by crystal symmetry. An approximate (isotropic) treatment of cell e.s.d.'s is used for estimating e.s.d.'s involving l.s. planes.

### (II) Diethyl 5,6-bis(pyridin-2-yl)pyrazine-2,3-dicarboxylate Fractional atomic coordinates and isotropic or equivalent isotropic displacement parameters (Å^2^)

**Table:**

	*x*	*y*	*z*	*U*_iso_*/*U*_eq_	
O1	0.7424 (2)	0.7559 (3)	0.00480 (5)	0.1009 (9)	
O2	0.91015 (17)	0.64422 (18)	−0.01674 (4)	0.0693 (5)	
N1	0.86590 (16)	0.73125 (18)	0.07318 (4)	0.0478 (5)	
N2	0.88149 (17)	0.8399 (2)	0.16340 (5)	0.0583 (6)	
C1	0.9332 (2)	0.7371 (2)	0.04156 (5)	0.0473 (5)	
C2	0.93113 (19)	0.7446 (2)	0.10488 (5)	0.0450 (5)	
C3	0.84753 (19)	0.7504 (2)	0.13824 (5)	0.0452 (5)	
C4	0.7388 (2)	0.6719 (2)	0.14134 (5)	0.0466 (5)	
H4	0.7174	0.6124	0.1225	0.056*	
C5	0.6613 (2)	0.6817 (2)	0.17249 (5)	0.0503 (5)	
H5	0.5874	0.6282	0.1755	0.060*	
C6	0.6952 (2)	0.7712 (2)	0.19868 (6)	0.0569 (6)	
H6	0.6448	0.7803	0.2202	0.068*	
C7	0.8042 (2)	0.8481 (3)	0.19322 (6)	0.0637 (7)	
H7	0.8256	0.9098	0.2114	0.076*	
C8	0.8508 (2)	0.7155 (3)	0.00796 (5)	0.0558 (6)	
C9	0.8402 (3)	0.6183 (4)	−0.05106 (7)	0.0978 (11)	
H9A	0.7744	0.5501	−0.0471	0.117*	
H9B	0.7956	0.6977	−0.0594	0.117*	
C10	0.9335 (4)	0.5760 (3)	−0.07867 (8)	0.0965 (11)	
H10A	0.8877	0.5552	−0.1013	0.145*	
H10B	0.9793	0.4990	−0.0699	0.145*	
H10C	0.9960	0.6455	−0.0833	0.145*	

### (II) Diethyl 5,6-bis(pyridin-2-yl)pyrazine-2,3-dicarboxylate Atomic displacement parameters (Å^2^)

**Table:**

	*U*^11^	*U*^22^	*U*^33^	*U*^12^	*U*^13^	*U*^23^
O1	0.0662 (13)	0.187 (2)	0.0499 (11)	0.0406 (14)	−0.0132 (9)	−0.0026 (12)
O2	0.0689 (11)	0.0889 (12)	0.0500 (9)	0.0023 (9)	−0.0168 (8)	−0.0172 (8)
N1	0.0424 (9)	0.0665 (12)	0.0347 (9)	0.0012 (8)	−0.0018 (7)	0.0026 (8)
N2	0.0464 (10)	0.0866 (14)	0.0418 (9)	−0.0104 (10)	0.0042 (8)	−0.0093 (9)
C1	0.0440 (10)	0.0630 (13)	0.0348 (10)	0.0039 (10)	−0.0018 (8)	0.0012 (9)
C2	0.0418 (10)	0.0581 (13)	0.0350 (10)	−0.0016 (9)	−0.0010 (8)	0.0011 (9)
C3	0.0379 (10)	0.0629 (13)	0.0348 (10)	0.0007 (9)	−0.0026 (8)	0.0026 (9)
C4	0.0421 (11)	0.0541 (12)	0.0437 (11)	0.0028 (9)	−0.0017 (8)	0.0041 (9)
C5	0.0407 (11)	0.0630 (13)	0.0471 (12)	0.0017 (10)	0.0035 (9)	0.0086 (10)
C6	0.0450 (12)	0.0826 (17)	0.0431 (11)	0.0052 (12)	0.0067 (9)	0.0062 (11)
C7	0.0553 (14)	0.0941 (18)	0.0416 (11)	−0.0080 (13)	0.0046 (10)	−0.0127 (12)
C8	0.0467 (13)	0.0851 (17)	0.0357 (11)	0.0035 (12)	−0.0008 (9)	0.0047 (11)
C9	0.095 (2)	0.141 (3)	0.0578 (16)	−0.007 (2)	−0.0290 (15)	−0.0268 (18)
C10	0.157 (3)	0.0720 (18)	0.0605 (17)	−0.020 (2)	−0.0135 (19)	−0.0139 (14)

### (II) Diethyl 5,6-bis(pyridin-2-yl)pyrazine-2,3-dicarboxylate Geometric parameters (Å, º)

**Table:**

O1—C8	1.189 (3)	C4—H4	0.9400
O2—C8	1.305 (3)	C5—C6	1.364 (3)
O2—C9	1.460 (3)	C5—H5	0.9400
N1—C2	1.337 (2)	C6—C7	1.379 (3)
N1—C1	1.339 (2)	C6—H6	0.9400
N2—C3	1.339 (3)	C7—H7	0.9400
N2—C7	1.343 (3)	C9—C10	1.450 (5)
C1—C1^i^	1.392 (4)	C9—H9A	0.9800
C1—C8	1.499 (3)	C9—H9B	0.9800
C2—C2^i^	1.413 (4)	C10—H10A	0.9700
C2—C3	1.483 (3)	C10—H10B	0.9700
C3—C4	1.377 (3)	C10—H10C	0.9700
C4—C5	1.384 (3)		
			
C8—O2—C9	117.3 (2)	C7—C6—H6	120.4
C2—N1—C1	118.40 (17)	N2—C7—C6	123.8 (2)
C3—N2—C7	116.06 (19)	N2—C7—H7	118.1
N1—C1—C1^i^	120.88 (11)	C6—C7—H7	118.1
N1—C1—C8	113.63 (18)	O1—C8—O2	124.2 (2)
C1^i^—C1—C8	125.48 (12)	O1—C8—C1	123.5 (2)
N1—C2—C2^i^	120.38 (11)	O2—C8—C1	112.27 (19)
N1—C2—C3	114.72 (17)	C10—C9—O2	108.7 (3)
C2^i^—C2—C3	124.88 (11)	C10—C9—H9A	109.9
N2—C3—C4	123.56 (18)	O2—C9—H9A	109.9
N2—C3—C2	115.71 (18)	C10—C9—H9B	109.9
C4—C3—C2	120.65 (18)	O2—C9—H9B	109.9
C3—C4—C5	119.1 (2)	H9A—C9—H9B	108.3
C3—C4—H4	120.4	C9—C10—H10A	109.5
C5—C4—H4	120.4	C9—C10—H10B	109.5
C6—C5—C4	118.2 (2)	H10A—C10—H10B	109.5
C6—C5—H5	120.9	C9—C10—H10C	109.5
C4—C5—H5	120.9	H10A—C10—H10C	109.5
C5—C6—C7	119.20 (19)	H10B—C10—H10C	109.5
C5—C6—H6	120.4		
			
C2—N1—C1—C1^i^	−3.1 (4)	C3—C4—C5—C6	1.1 (3)
C2—N1—C1—C8	177.7 (2)	C4—C5—C6—C7	0.1 (3)
C1—N1—C2—C2^i^	−4.4 (4)	C3—N2—C7—C6	−0.2 (4)
C1—N1—C2—C3	174.09 (19)	C5—C6—C7—N2	−0.6 (4)
C7—N2—C3—C4	1.6 (3)	C9—O2—C8—O1	3.0 (4)
C7—N2—C3—C2	178.3 (2)	C9—O2—C8—C1	−179.0 (2)
N1—C2—C3—N2	−137.7 (2)	N1—C1—C8—O1	37.9 (4)
C2^i^—C2—C3—N2	40.8 (4)	C1^i^—C1—C8—O1	−141.2 (3)
N1—C2—C3—C4	39.1 (3)	N1—C1—C8—O2	−140.1 (2)
C2^i^—C2—C3—C4	−142.4 (3)	C1^i^—C1—C8—O2	40.8 (4)
N2—C3—C4—C5	−2.1 (3)	C8—O2—C9—C10	162.0 (3)
C2—C3—C4—C5	−178.62 (19)		

Symmetry code: (i) −*x*+2, −*y*+3/2, *z*.

### (II) Diethyl 5,6-bis(pyridin-2-yl)pyrazine-2,3-dicarboxylate Hydrogen-bond geometry (Å, º)

Cg1 and Cg2 are the centroids of the pyrazine and pyridine rings N1/C1/C2/N1'/C1'/C2' and N2/C3–C7, respectively [symmetry code ('): -*x* + 2, -*y* + 3/2, *z*].

**Table:**

*D*—H···*A*	*D*—H	H···*A*	*D*···*A*	*D*—H···*A*
C7—H7···O1^ii^	0.94	2.48	3.308 (3)	147
C4—H4···*Cg*2^iii^	0.94	2.92	3.739 (2)	147
C10—H10*B*···*Cg*1^iv^	0.97	2.56	3.409 (3)	146
C10—H10*B*···*Cg*1^v^	0.97	2.56	3.409 (3)	146

Symmetry codes: (ii) −*y*+7/4, *x*+1/4, *z*+1/4; (iii) *y*−1/4, −*x*+5/4, −*z*+1/4; (iv) −*x*+2, −*y*+1, −*z*; (v) *x*, *y*−1/2, −*z*.
